# An ELISA to Detect Serum Antibodies to the Salivary Gland Toxin of *Ixodes holocyclus* Neumann in Dogs and Rodents

**DOI:** 10.1155/2011/283416

**Published:** 2011-05-18

**Authors:** S. Hall-Mendelin, P. O'Donoghue, R. B. Atwell, R. Lee, R. A. Hall

**Affiliations:** ^1^School of Chemistry and Molecular Biosciences, The University of Queensland, St. Lucia, QLD 4072, Australia; ^2^Public Health Virology, Queensland Health Forensic and Scientific Services, Coopers Plains, QLD 4108, Australia; ^3^School of Veterinary Science, The University of Queensland, St. Lucia, QLD 4072, Australia; ^4^Westmead Clinical School, The University of Sydney, NSW 2006, Australia

## Abstract

The *Ixodes holocyclus* tick causes paralysis in up to 10,000 companion and domestic animals each year in Australia. Treatment requires the removal of the parasite and the administration of a commercial tick antiserum that is prepared from hyperimmune dogs. Each batch of this serum is initially tested for toxin-neutralising potency in a mouse bioassay that is expensive, time consuming, and subjective. With the aim of developing a rapid *in vitro* assay to replace the bioassay, we used a partially purified antigen prepared from *I. holocyclus* salivary glands to develop an ELISA to detect toxin-reactive antibodies in hyperimmune dog sera. The optimised ELISA reliably detected antibodies reactive to *I. holocyclus* salivary gland antigens. Parallel testing of sera with a negative control antigen prepared from the salivary glands of the nontoxic tick *Rhipicephalus* (*Boophilus*) *microplus* provided further evidence that we were detecting toxin-specific antibodies in the assay. Using the ELISA, we could also detect antibodies induced in rats after experimental infestation with *I. holocyclus*. This assay shows promise as an alternative means of assessing the potency of batches of hyperimmune dog serum and to screen for toxin-reactive monoclonal antibodies produced from immunised rodents.

## 1. Introduction

Ixodid or hard ticks cause most toxicoses [1], affecting humans and animals around the world [2, 3]. The most severe form of toxicosis results in the paralysis of the infested host. Globally, just under 70 species of ticks have been described as being capable of inducing paralysis [2], the most important being *Ixodes holocyclus *in Australia, *Dermacentor andersoni*, *D. variabilis, *and *Argas *(*Persicargas*) *radiatus *in North America, *I. rubicundus *in South Africa, *Rhipicephalus evertsi evertsi *and *A. *(*P.*) *walkerae *in Ethiopia, and *A. *(*P.*) *radiatus *in the Nearctic region of North America [4]. 

In Australia, *I. holocyclus *can cause paralysis in a range of domestic animals and livestock [5], affecting up to 10,000 companion animals and up to 100,000 livestock per year [6]. It is considered highly toxic with one female able to kill a dog [1] or sheep [7]. 

*I. holocyclus *is found along the eastern seaboard of Australia and is most abundant from early spring to late summer. Paralysis is induced by a neurotoxin that is transmitted to the host in the saliva of a female *I. holocyclus *when the tick takes a blood meal. During feeding, toxicity in the salivary glands increases, peaking after 4-5 days of engorgement [8]. 

The treatment of paralysed hosts requires removal of the parasite and administration of a commercial tick anti-serum (TAS) that is prepared from hyperimmune dogs [9]. To standardise the toxin-neutralising activity of each batch of TAS, a mouse bioassay has been traditionally used. However, the bioassay is expensive, time consuming, and subjective, relying on observations of paralytic signs in neonatal mice over a 24-hour period [10]. Developing a suitable *in vitro* immunoassay to quantify toxin-specific antibodies in commercial TAS could replace the *in vivo* test. 

An *in vitro *assay may also facilitate timely diagnosis and inform on treatment options. Knowledge of the immune status of dogs before and after treatment for tick paralysis will help to evaluate a minimal, effective dose of TAS, avoiding possible adverse reactions and making treatment more affordable. 

In this paper, we describe the development of an enzyme-linked immunosorbent assay (ELISA) to measure antibody levels against tick salivary gland antigens in the serum of dogs and rodents after natural or laboratory tick infestation.

## 2. Materials and Methods

### 2.1. Preparation of Salivary Gland Antigens from Ticks

Ten adult unfed female *I. holocyclus* were allowed to feed on 10- to 11-week-old female Wistar rats following the methods of Stone et al. [11]. After 5 days, engorged ticks were carefully removed with tweezers, and salivary glands were excised and stored at −80°C. *Ixodes holocyclus* salivary glands were processed following the method of Stone et al. [10]. One hundred frozen salivary gland pairs were homogenised (Dounce, 7 mL) with 3 mL of sterile PBS and the homogenate clarified at 1500 g for 30 minutes at 4°C. The pellet was washed three times with 0.5 mL PBS and centrifuged as above. The pooled supernatant was sonicated to further disrupt remaining particulate matter with a Soniprep 150 MSE ultrasonicator using 30-second bursts followed by 2 minutes cooling on ice for a total of 10 minutes. The sonicated homogenate was pelleted at 109,000 g for 1 hour at 4°C, and the resulting supernatant was stored in aliquots at −80°C. Protein concentrations in antigen preparations were estimated with the BCA Protein Assay Kit as per protocol. All batches of *I. holocyclus* toxin were tested in the neonatal mouse bioassay to determine the level of paralysing activity [10].

### 2.2. Preparation of Control Antigen from the Cattle Tick *Rhipicephalus* (*Boophilus*) *microplus*

Salivary gland antigens from the cattle tick, *Rh. *(*B.*)* microplus,* which had fed for 5 days on cattle, were prepared in the same manner and used as a nontoxin control in all assays. These extracts were also tested in the bioassay, and no paralysis signs were observed in neonatal mice. 

### 2.3. Dog Serum Samples

A standardised reference serum was prepared by pooling several batches of commercially available TAS prepared from ten hyperimmune (HI) dogs and confirmed for toxin-neutralising activity in the mouse bioassay. Ten nonreactive dog sera were obtained from Perth in Western Australia, an area where *I. holocyclus* does not naturally occur. Sera were also obtained from dogs that presented at Manly Road Veterinary Hospital, Brisbane, for treatment against tick paralysis. Samples were collected at the time of admission, prior to treatment, and again approximately 16 days later.

### 2.4. Enzyme-Linked Immunosorbent Assay (ELISA)

An indirect ELISA format was employed for the analysis of sera. Various parameters were systematically optimised by empirical trials. The basic protocol was as follows: tick salivary gland antigen was diluted in coating buffer (0.05 M carbonate/bi-carbonate pH 9.6) and 50 *μ*L added to wells of ELISA plates (FALCON flexible, 96 wells, U bottom, Becton Dickinson) at 4°C overnight. After washing (phosphate-buffered saline containing 0.05% Tween 20) the plates twice to remove unbound antigen, 100 *μ*L of blocking buffer (0.05 M Tris, 0.001 M EDTA, 0.15 M NaCl, 0.2% w/v casein, 0.05% v/v Tween 20, pH 8.0) was applied to all wells for one hour at 37°C. After the removal of blocking buffer, 50 *μ*L of dog sera diluted in blocking buffer was added per well for 1 h at 37°C. After 4 washes, rabbit antidog IgG conjugated to horse-radish peroxidase (whole molecule, HRPO, SIGMA) was diluted in blocking buffer, added (50 *μ*L per well), and allowed to bind to captured dog IgG for a further hour at 37°C. After an additional 6 washes, bound conjugate was detected with 100 *μ*L of ABTS substrate (2% of 2,2′-Azino-bis (3-ethylbenzo-thiazoline-6-sulfonic acid) diammonium salt, SIGMA; 2% of 1.26% H_2_O_2_ in substrate buffer: 2.8% Na_2_HPO_4_ and 2.1% citric acid, pH 4.2). After a 1-h reaction at 37°C, the spectroscopic absorbance of each well was measured in an automated plate reader (MULTISKAN) at a wavelength of 405 nm.

### 2.5. Optimisation of ELISA to Detect Toxin-Reactive Antibodies in Rat Sera

A modified ELISA was used to test sera from rats experimentally infested with *I. holocyclus* ticks. Serum was collected from rats prior to and at various times after ticks had fed on the animals for five days. Pre- and post- tick infestation sera were then tested in the ELISA. In the absence of rat serum known to be reactive to tick toxin, the optimised protocol for dog sera was employed, with the exception that horseradish peroxidase-conjugated antirat IgG diluted 1 : 1000 was used for the second antibody step.

## 3. Results

### 3.1. Optimisation of ELISA Parameters

Initial analysis of HI dog sera revealed that high levels of nonspecific binding to the plastic in the wells of the ELISA plate occurred at dilutions below 1/50 (results not shown). Therefore, all dog sera were subsequently tested at dilutions of 1/50 or greater. 

**Figure 1:**
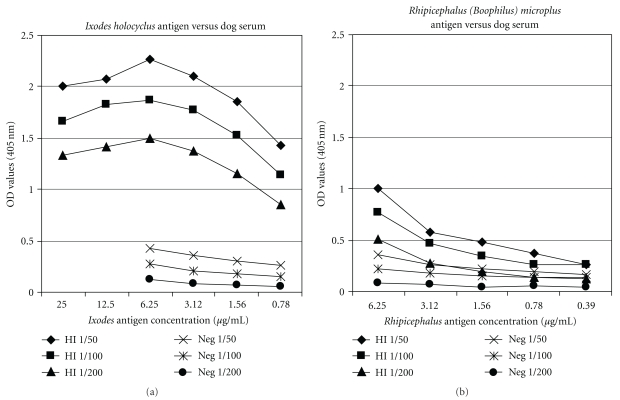
Optimisation of salivary gland antigen concentration in ELISA. Doubling dilutions (1/50–1/200) of pooled hyperimmune (HI) or negative (Neg) dog sera were incubated with varying concentrations of *Ixodes *(a) or *Rhipicephalus* (b) antigen in ELISA. Antigens were diluted in carbonate/bicarbonate coating buffer and adsorbed to the solid phase at 4°C overnight. All other steps were performed at 37°C for 1 h as described in Section 2.

To determine the optimal dilution of *I. holocyclus *salivary gland antigen, pooled HI dog serum and antigen were titrated in a checkerboard format. Serum dilutions of 1/50–1/200 demonstrated maximum binding to *Ixodes* antigen at 6.25 *μ*g/mL with optical densities (OD) between 1.5 and 2.3 (Figure 1(a)). Negligible binding was observed for each dilution of the negative control dog serum at all antigen concentrations examined (OD < 0.5). Therefore, 6.25 *μ*g/mL was chosen as the antigen concentration for all subsequent assays. 

HI and negative dog sera were also assayed against similar concentrations of salivary gland antigen prepared from the cattle tick, *Rh. *(*B.*)* microplus* (Figure 1(b)). All sera dilutions showed significantly reduced or negligible binding to this antigen compared to similar concentrations of antigen prepared from *Ixodes* salivary glands indicating that most of the ELISA-reactive antibody in the sera was specific to the *Ixodes *antigen. Some cross-reactivity was observed for the lowest dilutions (1/50 and 1/100) of HI dog (ODs between 0.5 and 1.0) with the *Rh. *(*B.*)* microplus* antigen at the two highest antigen concentrations (3.12 and 6.25 *μ*g/mL) consistent with the recognition of salivary gland antigens common to both *I. holocyclus* and *Rh. *(*B.*)* microplus. *In summary, these results demonstrate that partially purified tick salivary gland antigens containing *I. holocyclus* toxin are specifically recognised by antibodies in hyperimmune dog sera in ELISA. 

To determine whether antigen adsorbtion could be improved by various combinations of coating buffers, incubation temperatures, and times, *I. holocyclus* or *Rh. *(*B.*)* microplus* antigens were diluted in carbonate/bicarbonate buffer (pH 9.6) or PBS (pH 7.2) and incubated in ELISA plate wells at 4°C overnight or 37°C for 3 h. When ELISA plates were then tested against HI and negative dog sera titrated from 1/50 in blocking buffer, antigens adsorbed in coating buffer at 37°C resulted in significantly higher binding of both HI and negative control serum antibodies to both *I. holocyclus* and *Rh. *(*B.*)* microplus* antigens compared with adsorption at 4°C (results not shown). The best differential binding between HI and negative serum on *I. holocyclus* antigens and HI serum on *I. holocyclus* and *Rh. *(*B.*)* microplus* antigens was achieved by adsorbing antigens in carbonate coating buffer at 4°C (results not shown).

### 3.2. Establishing a Cutoff OD Value to Distinguish between Positive and Negative Reactions in Dog Sera

**Figure 2:**
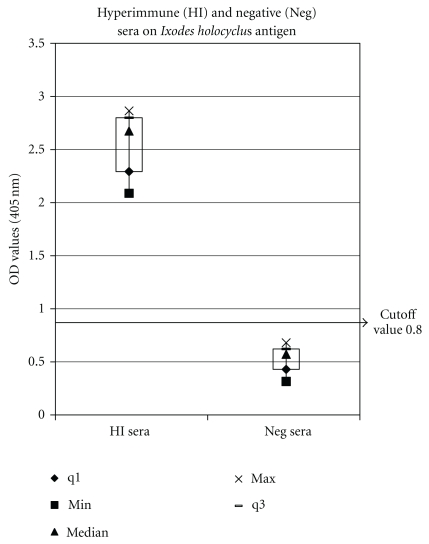
Reactivity of 10 HI and 10 negative dog serum samples tested in ELISA shown in a Box and Whisker diagram: q1 lower quartile, cuts off lowest 25% of data; q3 third quartile, cuts of highest 25% of data.

A panel of ten HI and ten negative sera at 1/50 was tested in the ELISA using the optimal conditions described above. The results are presented in a box and whisker diagram (Figure 2), illustrating the spread of data. The data set is divided into four equal parts, the quartiles, each representing 25% of the data. The lower quartile (q1) encompasses the lowest 25% of reactors and the upper quartile (q3) the highest 25% of reactors. The median is the middle value with 50% of the data below and above. Minimum (min) is the lowest value, and maximum (max) is the highest value of the data set. 

The cutoff value for negative sera tested against *Ixodes* antigen was calculated as the mean of ten negative sera (0.525) plus twice the standard deviation (2 × 0.14) = 0.805.

### 3.3. Analysis of Sera from Dogs Naturally Infested with *I. holocyclus* and Suffering Tick Paralysis

Eight dogs (A to H) presented at the Manly Road Veterinary Hospital with signs of tick paralysis. On day 0 (upon presentation and prior to treatment) only sera from dogs C, D, and H reacted strongly with *Ixodes* antigen when tested in the ELISA (OD > 1.0) (Table 1). Only the day 0 sera from dog C showed significantly less reactivity with *Rhipicephalus* antigen, while the corresponding sample from dogs D and H reacted similarly with both antigens. Antibody levels to *Ixodes* antigen from the other five dogs fell below the cutoff value determined earlier. Testing of serum samples from day 16 (after administration of TAS) revealed a sharp increase of antibody levels to *Ixodes* antigen in dogs C and H, while a similar increase of antibody levels to *Rhipicephalus* antigen was also observed in dog C. Antibodies in sera from dog G did not react with *Ixodes* antigen on day of admission but rose just above the cutoff threshold on day 16, after treatment with TAS. Antibody levels from all other dogs did not rise above the levels from day 0. These results are summarised in Table 1.

### 3.4. Optimisation of ELISA for Testing Immune Rat Sera

Serum from seven rats taken prior to and 21 days after infestation with ticks was tested in an ELISA modified to detect rat immunoglobulin to tick toxin. All samples tested on *Ixodes *antigen in ELISA showed slightly increased antibody levels in postinfestation samples as compared to preinfestation serum, although OD levels remained well below values seen with HI dog sera. ODs for *Rhipicephalus* antigen did not increase in three rats while increased only marginally in the remaining four rats (see Table 2). These preliminary data indicated that we could detect, albeit weakly, antibodies to the toxin-associated antigen in rat sera after a single infestation with multiple ticks.

**Figure 3:**
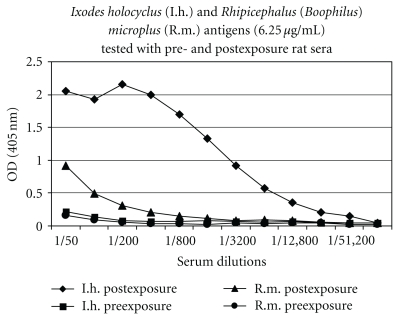
The reaction of rat sera to *Ixodes* antigen in ELISA after two exposures to *I. holocyclus* ticks. Preexposure (negative) and postexposure (positive) rat serum was titrated in doubling dilutions and tested against *Ixodes* and *Rhipicephalus* antigen in ELISA using the optimal conditions and protocol described earlier.

Further analysis of the seroconversion of a rat after sequential infestations with multiple ticks was also undertaken. Serum collected from this animal after two rounds of infestations over 248 days exhibited strong, specific reaction to *Ixodes* antigen when tested in ELISA compared to serum taken prior to infestation (see Figure 3). 

Collectively, these results reveal that the ELISA can be used to detect serum antibodies specific for *Ixodes* antigen, induced by single or multiple exposures to *I. holocyclus* ticks.

## 4. Discussion

A partially purified antigen preparation from the salivary glands of female *I. holocyclus* ticks was assessed for use as a diagnostic reagent in ELISA to measure tick toxin-specific antibodies in dog sera. The results from these assays indicated that sera from HI dogs reacted strongly to the *Ixodes *antigen, compared to negligible reactions of sera from dogs not exposed to *I. holocyclus* ticks. Furthermore, a control antigen prepared from the salivary glands of the cattle tick *Rh. *(*B.*)* microplus*, which does not produce paralysis toxin, showed relatively weak reactions for both HI (positive) and control (negative) sera. The reaction of some dog sera to antigen from *Rh. *(*B.*)* microplus* seen in ELISA was to be expected due to common antigens in the salivary glands in both tick species. Indeed, amino acid sequences between *Ixodes* and *Rhipicephalus* (*Boophilus*) ticks show a high degree of homology, >85% [12] indicating a close antigenic relationship. These data suggest that the ELISA detected antibodies specific to toxin-associated antigens and that *in vitro* analysis of commercially prepared TAS may be a viable alternative to the expensive and subjective mouse bioassay.

Previously, the Australian Pesticides and Veterinary Medicines Authority (APVMA) has declared that an *in vitro* immunoassay to evaluate commercial TAS would only be approved if a highly specific antigen was used (B. Stone, pers comm.). Indeed, Morrison (unpublished data) [13] used a partially purified antigen extracted from *I. holocyclus* salivary glands in an ELISA and claimed good correlation with the bioassay. However, in the absence of a control antigen (a similar extract from toxin-free ticks), the author was unable to demonstrate the reaction to the antigen was toxinspecific. 

In contrast to the strong reactivity of HI dog sera in the ELISA, an apparent lack of antibody responsiveness was observed in sera from dogs naturally infested with ticks and presenting with paralysis signs at a Brisbane veterinary clinic. These sera were tested in the ELISA to assess the level of toxin-specific antibodies at the onset of signs and approximately 16 days later. According to ELISA OD readings, three out of eight dogs showed significant antibodies to tick toxin antigen at the time of presentation, but only two of seven exhibited a significant rise in antitick antibody 16 days later. A fourth serum was negative to *Ixodes* antigen on presentation but seroconverted after treatment to just above threshold level. In the absence of additional information on the general health status of the dogs before and after treatment, including weight, breed, sex, age, history of previous tick exposure, and the dosage and batch of TAS used, interpretation of the data was limited. However, based on the observation that at least half (4/7) of the animals showed no increase in serum antibody levels to the *Ixodes* antigen after treatment with TAS, we may conclude that the increased ELISA response to this antigen detected in some animals was more likely due to an immune response to tick infestation rather than the passive transfer of antibodies through the TAS treatment. 

Based on the low rate of seroconversion to toxin-specific antigens in dogs naturally exposed to *I. holocyclus*, even after treatment with TAS, it is unlikely that the assay will be a useful tool for clinicians to monitor the immune status of animals or the efficacy of treatment. This is consistent with findings of Stone and Wright [14] who observed slow and infrequent neutralising antibody responses (measured in a mouse bioassay) in dogs over 14 weeks of repeated tick infestation during the priming phase towards hyperimmunity. The lack of immune response is likely due to the equilibrium achieved between host immunity to the tick and the tick's suppression of the host's immune system. The feeding tick modulates the host's haemostasis, inflammation, and immunity [15] to enable uninterrupted engorgement. This is supported by the fact that *I. holocyclus* ticks are not rejected (no weight loss) and dogs show very little cutaneous hypersensitivity despite repeated exposure [16]. 

In summary, an ELISA was established which differentiated strongly between HI dog sera and sera from dogs that had not been infested with *I. holocyclus* ticks. Since the *Ixodes* salivary gland antigen was only partially purified, the specificity of this ELISA was supported by parallel testing of the sera on an antigen prepared in the same way from nontoxic *Rhipicephalus* ticks. HI and nonreactive sera bound substantially weaker to the *Rhipicephalus* antigen, suggesting that reactions of HI sera to the *Ixodes* antigen were specific. Sera from dogs experimentally or naturally infested with a limited number of ticks, however, produce only weak reactions to the *Ixodes* antigen in ELISA, suggesting that antibody levels to low-frequency tick infestations are not detected in this assay. 

In the absence of highly purified toxin antigen, the specificity of the assay may be enhanced by the use of tick toxin-reactive monoclonal antibodies to capture toxin-specific antigen to the solid phase in a modified ELISA format. To this end, the ELISA described above was successfully adapted for the detection of antibodies in rats exposed to tick infestation. Indeed, the detection of strong antibody responses in rats after two exposures to ticks indicates that this animal model will be useful for producing hybridomas to* I. holocyclus* toxin antigens and the ELISA an effective means for screening the resultant monoclonal antibodies.

## Figures and Tables

**Table 1:** Reactions of sera from dogs suffering from tick paralysis to *Ixodes holocyclus* (I.h) and *Rhipicephalus* (*Boophilus*) *microplus *(R.m) antigen in ELISA.

	Day 0^1^		Day 16^2^	
Dog ID	I.h	R.m	I.h	R.m
A	0.666^3^	0.518	n/a	n/a
B	0.536	0.477	0.515	0.443
C	1.227	0.706	2.223	1.358
D	1.902	1.13	1.769	0.993
E	0.41	0.348	0.43	0.359
F	0.538	0.335	0.536	0.363
G	0.565	0.318	0.828	0.362
H	1.047	1.05	2.068	1.201

^1^Sera were collected on day of admission prior to the administration of TAS.

^2^Sera were collected approximately 16 days after TAS was administered.

^3^Optical density (405 nm) of ELISA reaction of sera (1/50) to *Ixodes* (I.h) and *Rhipicephalus* (R.m) antigen.

**Table 2:** The reaction of sera from rats exposed to *Ixodes holocyclus* ticks with *Ixodes holocyclus* and *Rhipicephalus* (*Boophilus*) *microplus* antigens in ELISA.

Rat ID	OD_(405 nm)_ ELISA			
	*Ixodes antigen*		*Rhipicephalus antigen*	
	Preinfestation*	Postinfestation*	Preinfestation	Postinfestation
A	0.19 ± 0.04	0.29 ± 0.005	0.08 ± 0.002	0.07 ± 0.005
B	0.15 ± 0.02	0.31 ± 0.004	0.11 ± 0.003	0.15 ± 0.007
C	0.2 ± 0.01	0.25 ± 0.004	0.13 ± 0.06	0.05 ± 0.004
D	0.2 ± 0.05	0.4 ± 0.002	0.08 ± 0.002	0.16 ± 0.001
E	0.11 ± 0.03	0.2 ± 0.007	0.08 ± 0.006	0.08 ± 0.007
F	0.14 ± 0.02	0.25 ± 0.005	0.068 ± 0005	0.14 ± 0.003
G	0.17 ± 0.06	0.3 ± 0.005	0.1 ± 0.004	0.12 ± 0.003

Tick Naïve Rat	0.11 ± 0.03	0.09 ± 0.001	0.11 ± 0.004	0.07 ± 0.004

*Sera were collected from individual rats before and 21 days after tick infestation, diluted 1/50, and tested against *Ixodes* and *Rhipicephalus* antigens in ELISA using the optimised conditions and protocol described earlier.
